# Changes in the Proteome Profile of People Achieving Remission of Type 2 Diabetes after Bariatric Surgery

**DOI:** 10.3390/jcm10163659

**Published:** 2021-08-18

**Authors:** Zohaib Iqbal, Helene A. Fachim, J. Martin Gibson, Ivona Baricevic-Jones, Amy E. Campbell, Bethany Geary, Rachelle P. Donn, Dashne Hamarashid, Akheel Syed, Anthony D. Whetton, Handrean Soran, Adrian H. Heald

**Affiliations:** 1 The School of Medicine and Manchester Academic Health Sciences Centre, Manchester University, Manchester M13 9PL, UK; zohaib@doctors.org.uk (Z.I.); martin.gibson@manchester.ac.uk (J.M.G.); Rachelle.donn@manchester.ac.uk (R.P.D.); handrean.soran@mft.nhs.uk (H.S.); 2 Department of Endocrinology, Diabetes and Metabolism, Salford Royal Foundation Trust, Salford M6 8HD, UK; hamarashiddashne@gmail.com (D.H.); akheel.syed@manchester.ac.uk (A.S.); 3 Stoller Biomarker Discovery Centre, Division of Cancer Sciences, School of Medical Sciences, Faculty of Biology, Medicine and Health, University of Manchester, Manchester M13 9PL, UK; ivona.baricevic-jones@manchester.ac.uk (I.B.-J.); amy.campbel@manchester.ac.uk (A.E.C.); bethany.geary@manchester.ac.uk (B.G.); tony.whetton@manchester.ac.uk (A.D.W.); 4 Manchester National Institute for Health Research Biomedical Research Centre, Manchester M13 9WL, UK

**Keywords:** bariatric surgery, SWATH-MS, proteomics, diabetes, remission

## Abstract

Bariatric surgery (BS) results in metabolic pathway recalibration. We have identified potential biomarkers in plasma of people achieving type 2 diabetes mellitus (T2DM) remission after BS. Longitudinal analysis was performed on plasma from 10 individuals following Roux-en-Y gastric bypass (*n* = 7) or sleeve gastrectomy (*n* = 3). Sequential window acquisition of all theoretical fragment ion spectra mass spectrometry (SWATH-MS) was done on samples taken at 4 months before (baseline) and 6 and 12 months after BS. Four hundred sixty-seven proteins were quantified by SWATH-MS. Principal component analysis resolved samples from distinct time points after selection of key discriminatory proteins: 25 proteins were differentially expressed between baseline and 6 months post-surgery; 39 proteins between baseline and 12 months. Eight proteins (SHBG, TF, PRG4, APOA4, LRG1, HSPA4, EPHX2 and PGLYRP) were significantly different to baseline at both 6 and 12 months post-surgery. The panel of proteins identified as consistently different included peptides related to insulin sensitivity (SHBG increase), systemic inflammation (TF and HSPA4—both decreased) and lipid metabolism (APOA4 decreased). We found significant changes in the proteome for eight proteins at 6- and 12-months post-BS, and several of these are key components in metabolic and inflammatory pathways. These may represent potential biomarkers of remission of T2DM.

## 1. Introduction

Obesity is a chronic disease which in many cases requires complex management, while being recognized as the fastest growing problem affecting public health worldwide [1,2,3].

Obesity often has multiple predisposing and precipitating factors and is itself associated with a high mortality rate and with comorbid conditions such as type 2 diabetes mellitus (T2DM), metabolic syndrome, hypertension, dyslipidemia, several cancers [4], premature cell ageing [5], sleep apnea and osteoarthritis [6,7]. Furthermore, obesity is a risk factor for dementia and mild cognitive impairment [8].

Bariatric surgery (BS) has been shown not only to be an effective therapy for weight loss, but also improves a variety of metabolic parameters conferring protection from cardiovascular (CVD) and other diseases [9,10]. Immediate post-operative weight-independent effects combined with weight loss can result in remission of T2DM in up to 80% of patients [10]. Remission is observed also in patients undergoing gastrointestinal surgery similar in design to BS for other reasons such as gastric cancer, which reportedly has better T2DM remission rates [11]. Normalization of glucose metabolism occurs through mechanisms that have not yet been fully determined [11,12]. However, different studies have shown that reductions in waist circumference after BS are associated with a greater probability of T2DM remission [13,14,15].

The study of proteins and their functions has been important in helping investigators to decipher the cellular mechanisms that relate to particular phenotypes [16,17], in addition to accelerating the search for biomarkers for predicting disease pathogenesis and outcomes.

Sequential window acquisition of all theoretical fragment ion spectra mass spectrometry (SWATH-MS) is a data independent acquisition method, which aims to enable larger clinical studies by offering relative quantification across multiple samples [18]. It allows a complete and permanent recording of all fragment ions of the detectable peptide precursors present in a biological sample [19].

In order to have a wider view of the changes that occur after weight loss induced by BS as they relate to remission of T2DM and begin to understand if there are potential early biomarkers for T2DM remission, we set out to identify the changes in the plasma proteome of T2DM patients following BS.

## 2. Materials and Methods

Ten individuals (2 males and 8 females) were selected from the subset of those who had achieved remission of T2DM following Roux-en-Y gastric bypass (*n* = 7) or sleeve gastrectomy (*n* = 3) BS as participants in a larger prospective study (please see Adam et al. for further details of methodology [20]). All were of Caucasian ethnicity. Patients were selected on the basis they had achieved remission of T2DM at 12 months. Remission was defined as reduction of glycosylated haemoglobin (HbA1c) below 42 mmol/mol with cessation of all anti-diabetic therapy at 12 months [21]. SWATH-MS proteomics was carried out on 29 plasma samples with the following sample sub-groups: baseline samples, pre-bariatric surgery (taken at 4 months prior to BS (*n* = 10); post-bariatric surgery samples, 6 months follow up (*n* = 10); post-bariatric surgery samples, 12 months follow up (*n* = 9).

### 2.1. Sample Preparation for SWATH-MS

Plasma samples (10 µL) were immunodepleted using top 12 abundant protein depletion spin columns from Pierce Biotechnology, UK. Depleted plasma was buffer exchanged and concentrated using Amicon centrifugal filters. Total protein concentration was determined by microplate format (bicinchoninic acid assay). Each sample was reduced, alkylated and digested prior to lyophilisation. More details available in McGurk et al., 2020 [22].

### 2.2. Liquid Chromatography Mass Spectrometry (LC-MS) Instrument Analyses

Samples were analysed by SWATH-MS with a micro-flow LC-MS system comprising an Eksigent nanoLC 400 autosampler and an Eksigent nanoLC 425 pump coupled to a SCIEX 6600 Triple-TOF mass spectrometer with a DuoSpray Ion Source. The system was configured for a trap-elute elute analysis in which sample was injected from the autosampler (8 °C) onto a trap column (YMC- Triart C18; length: 5 mm; ID: 0.5 mm; particle size: 3 µm; pore size: 120 Å) with loading buffer mobile phase (10 µL min-1, 3 min, 2% acetonitrile, 0.1% formic acid) then eluted through an analytical column (YMC-Triart C18; length: 150 mm; ID: 0.3 mm; particle size: 3 µm; pore size: 120 Å; 30 °C) with the required analytical gradient into the mass spectrometer source. The system was controlled by Analyst software v1.7.1 and Eksigent control software v4.2 (SCIEX, Nieuwerkerk aan den IJssel, The Netherlands).

Peptides were eluted with an analytical gradient (pick-up 10 µL, 5 µL/min, 68 min) and analysed using a mass spectrometer method with a total duty cycle of 2.59 s comprising a TOF MS1 scan (*m*/*z* 400–1250, 250 ms) followed by 100 SWATH-MS scans (*m*/*z* 100–1500, 20 ms) with variable *m*/*z* isolation widths, collision energy and collision energy spread. SWATH maps generated via microflow LC on line to mass spectrometry for 68 min (as described in reference [23]). SWATH-MS data were searched using openSWATH (Version 2.0.0) against the Stoller human serum spectral library. Peptide matches were scored using pyProphet (Version 0.18.3) and search results were aligned using the feature alignment script from MSproteomicstools. Downstream analysis was performed in R using the bioconductor (release 3.5) packages SWATH2Stats and MSstats.

### 2.3. Determination of Glycosylated Haemoglobin, Glucose and Insulin

HbA1c was measured on an Hb9210 Premier autoanalyser (boranate affinity and high-performance liquid chromatography (Menarini Diagnostics, Wokingham, Berkshire, UK). Glucose and insulin were measured using Abcam ELISA kits (Abcam, Cambridge, UK) and using the HOMA2 calculator (https://www.dtu.ox.ac.uk/homacalculator/ accessed on 2 May 2020) were used to calculate the homeostatic model assessment of insulin resistance (HOMA-IR) and beta cell function (HOMA-B) [24].

### 2.4. Ethics

Informed consent was obtained from each participant before recruitment. This research adhered to the tenets of the Declaration of Helsinki. All participants provided written informed consent. The study was approved by the Greater Manchester Research Ethics Committee (REC No:11/NW/0731, IRAS ID: 85208)

## 3. Statistical Analysis

Statistical analysis of anthropometric and biochemical measurements was carried out on SPSS for Mac (Version 23.0, IBM Corporation, New York, NY, USA). Normality was determined by using the Shapiro-Wilk test and by visualising the histogram and normal Q-Q plot. To assess within and between group differences we used one-way analysis of variance for parametric variables and Friedman’s test for non-parametric variables. A significant *p* value was considered to be <0.05 (post hoc—Tukey). Statistical analysis was performed using all proteins quantified at both time points in 3 or more individuals; 6 months vs. baseline = 314 proteins; 12 months vs. baseline = 317 proteins; 6 months vs. 12 months = 309 proteins.

Principal component analysis (PCA) and heatmaps with hierarchical clustering (Euclidean distance) were plotted to assess separation between time points. Differential expression analysis was performed using the LIMMA package in R (version 3.46.0) on protein fold changes calculated for each of the 10 individuals at 6- or 12-months post-surgery relative to pre-surgery samples, taking a *p*-value ≤ 0.05 as significant.

## 4. Results

Longitudinal sampling is of value in biomarker analyses. Thus, the anthropometric and biochemical measurements were taken at baseline, 6- and 12-months as shown in Table 1 and SWATH-MS data described below. Two men and 8 women with a mean age of 53 years were included in the analysis. Seven individuals underwent Roux-en-Y gastric bypass and three underwent sleeve gastrectomy.

BS resulted in a significant reduction in body mass index (BMI) after 6- and 12-months (*p* < 0.0001), systolic and diastolic blood pressure after 12 months (*p* < 0.05) but not 6 months, HbA1c after 6- and 12-months (*p* < 0.0001). Fasting plasma glucose declined significantly at both 6 and 12 months (*p* < 0.05). HOMA2-IR declined at 6 and 12 months but did not reach significance (*p* = 0.57) and HOMA2-B (%) increased at 6 and 12 months (*p* = 0.10) but this also did not reach significance (*p* = 0.14) (Table 1).

**Figure 1:**
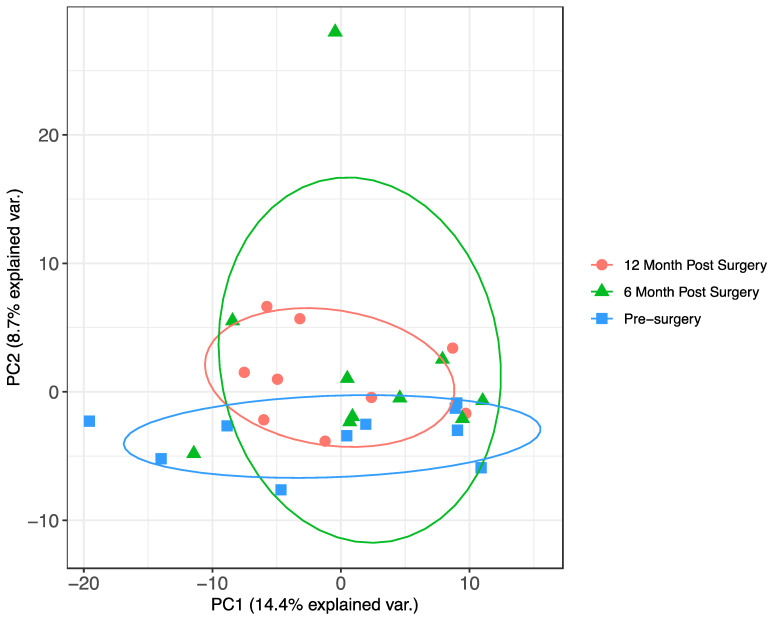
Principal component analysis of all identified proteins. Pre-surgery samples are coloured in blue, 6-month post-surgery samples in green and 12-month post-surgery samples in red.

**Figure 2:**
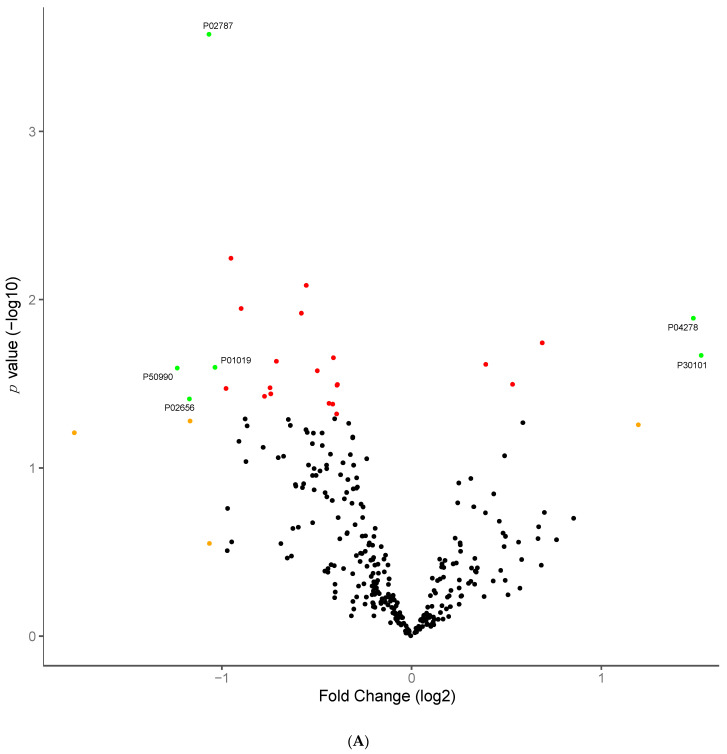

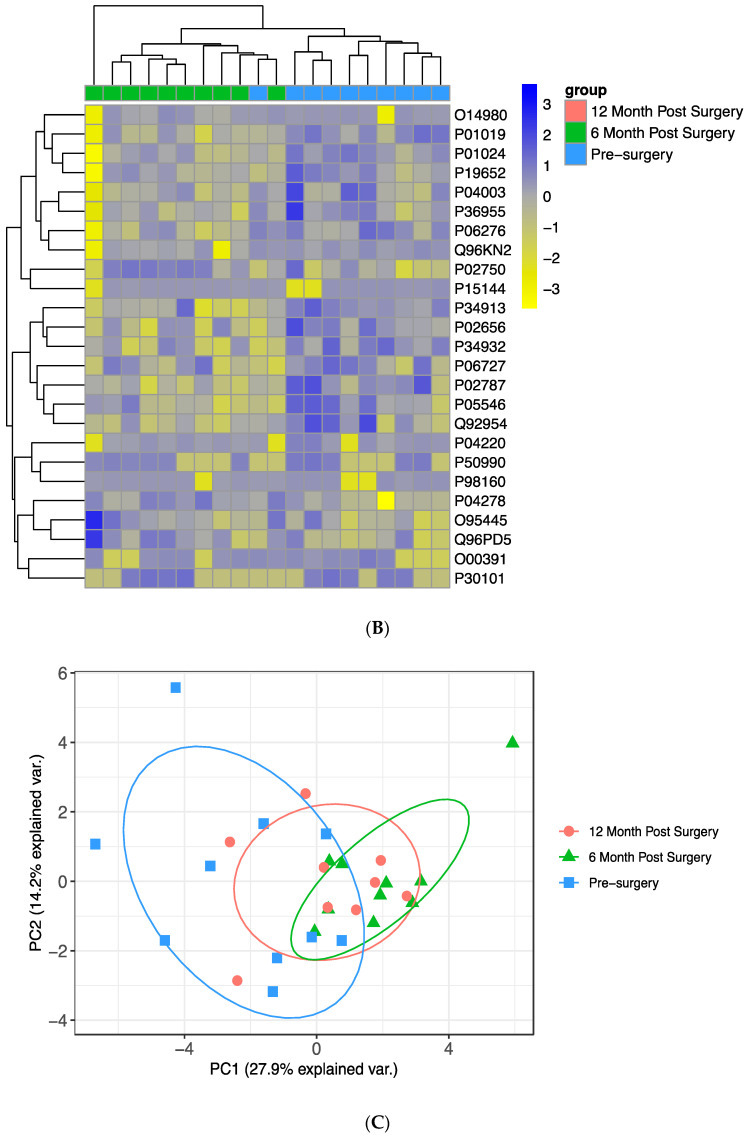
Protein abundance changes 6 months post-surgery. (**A**) Volcano plot: green points are significant and have a fold change >1, red points are significant but have a fold change <1 and black points are not significant. Protein abundance changes 6 months post-surgery. (**B**) Hierarchical clustering and heatmap of all 25 proteins significantly different 6 months post-surgery relative to pre-surgery samples. Hierarchical clustering was performed using the Euclidean distance. Row scaling was performed on the log2 abundance for each protein by the subtraction of the mean from each feature and then dividing by the standard deviation. Protein abundance changes 6 months post-surgery (**C**) Principal component analysis with all 25 proteins significantly different 6 months post-surgery relative to pre-surgery samples.

All 10 individuals underwent remission of T2DM achieving HbA1c less than 42 off all diabetes medications after 12 months. Following SWATH-MS analysis, principal component analysis (PCA) revealed very little separation between the different time points when including all 467 quantified proteins (Figure 1). Using differential expression analysis, 25 proteins were significantly different between pre-surgery and 6 months post-surgery samples (Table 2, Figure 2A). These include three apolipoproteins (APOC3, APOM and APOA4), sex hormone binding globulin (SHBG), serotransferrin (TF) and angiotensinogen (AGT). Hierarchal clustering with log2 protein abundances scaled across all pre-surgery and 6 months post-surgery samples shows that, with just the 25 significant proteins, it is possible to discriminate 6 months post-surgery from pre-surgery samples (Figure 2B). PCA using log2 protein abundance ratios for each of the 25 discriminatory proteins across all 10 individuals, again shows that the significant proteins largely separate pre-surgery from 6-month post-surgery samples, with the 12-month post-surgery samples appearing more similar to the 6-month post-surgery samples (Figure 2C). Gene ontology (GO) term enrichment analysis of the 25 discriminatory proteins showed significant enrichment for proteins involved in 55 main biological processes, among them and most important to this context: response to stimulus, leukocyte mediated immunity, plasma lipoprotein remodelling and protein-lipid complex remodelling pathways. All pathways displayed had a Benjamini adjusted *p*-value ≤ 0.05 and a minimum of 3 proteins contributing to the enrichment. The discriminatory proteins with the biggest fold enrichment (FE) changes were APOA4, APOC3, APOM and AGT (FE = 96.35; *p* = 0.002).

**Figure 3:**
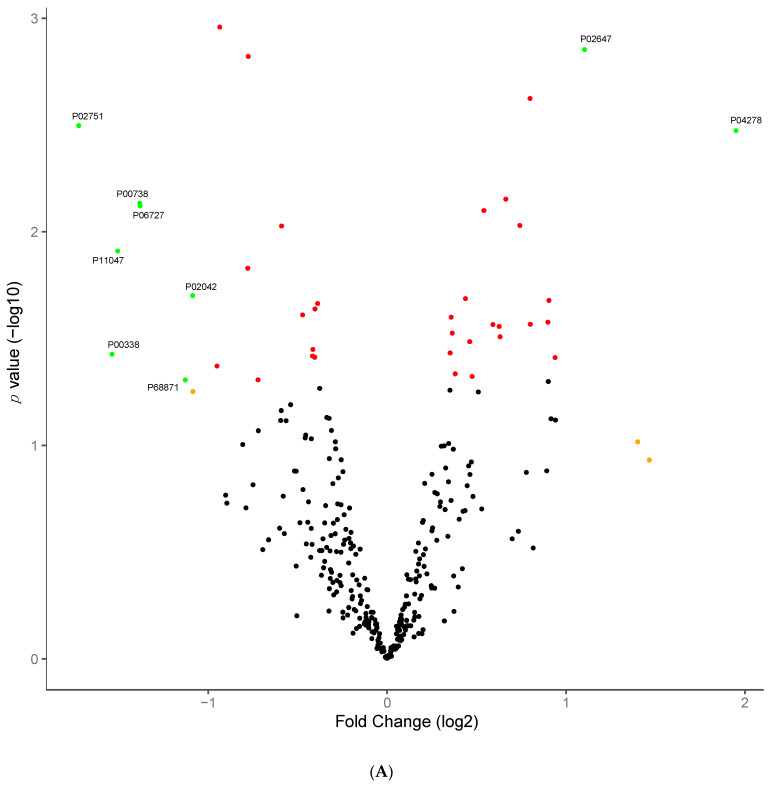

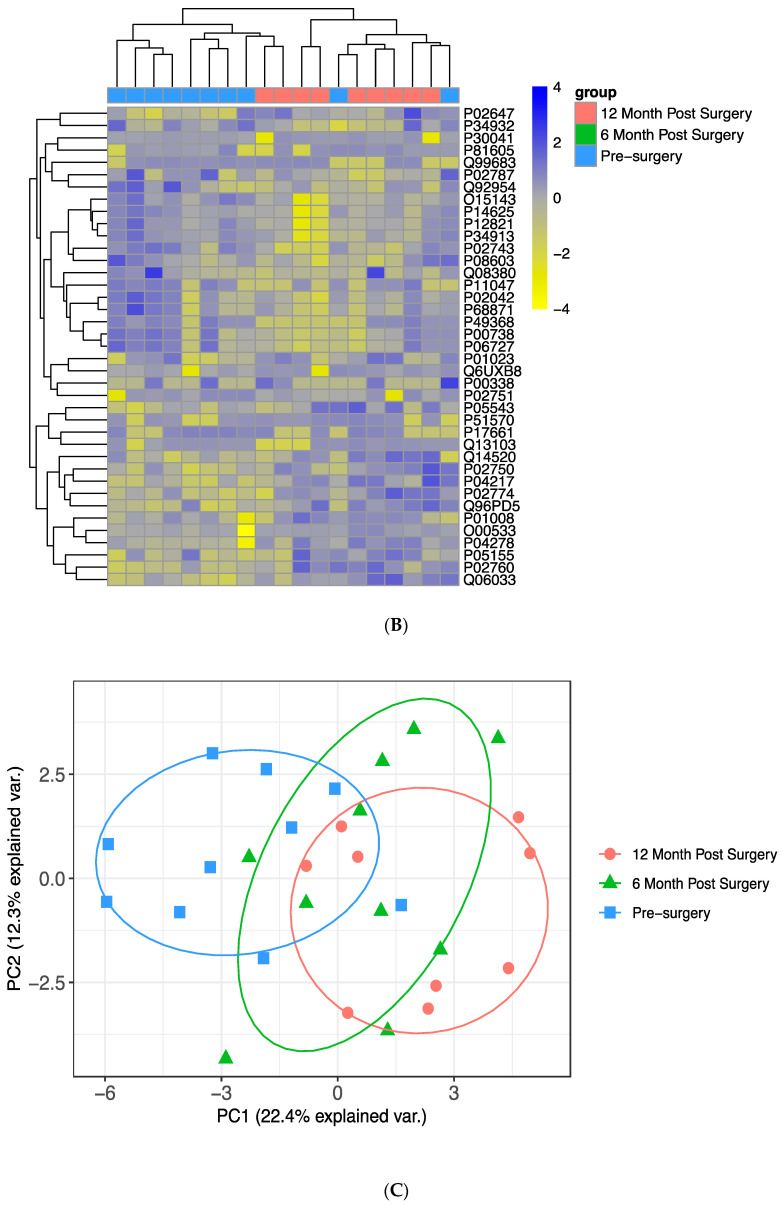
Protein abundance changes 12 months post-surgery. (**A**) Volcano plot: green points are significant and have a fold change >1, red points are significant but have a fold change <1 and black points are not significant. Protein abundance changes 12 months post-surgery. (**B**) Hierarchical clustering and heatmap of all 39 proteins significantly different 12 months post-surgery relative to pre-surgery samples. Hierarchical clustering was performed using the Euclidean distance. Row scaling was performed on the log2 abundance for each protein by the subtraction of the mean from each feature and then dividing by the standard deviation. Protein abundance changes 12 months post-surgery. (**C**) Principal component analysis with all 39 proteins significantly different 12 months post-surgery relative to pre-surgery samples.

When comparing pre-surgery samples to those collected 12 months post-surgery there was a greater degree of difference, with 39 proteins significantly different (Table 3, Figure 3A). These include two apolipoproteins (APOA1 and APOA4), SHBG, fibronectin (FN1) and haptoglobulin (HP). With those 39 significant proteins, hierarchical clustering of scaled log2 protein abundances showed discrimination between pre-surgery and 12 months post-surgery samples (Figure 3B). PCA with log2 protein abundance ratios for the 39 discriminatory proteins shows that a greater degree of separation was achieved at 12 months post-surgery, with samples taken 6 months post-surgery falling in between the pre-surgery and 12-month samples based on these 39 discriminatory proteins (Figure 3C). GO term enrichment analysis of the 39 discriminatory proteins showed enrichment for proteins involved in 86 significant biological processes, most of them involving healing, blood coagulation and immune activation pathways. All pathways displayed had a Benjamini adjusted *p*-value ≤ 0.05 and a minimum of 3 proteins contributing to the enrichment. The proteins showing the biggest FE were: Antithrombin-III (SERPINC1), Plasma protease C1 inhibitor (SERPING1), Alpha-2-macroglobulin (A2M) and FN1 (FE = 69.34; *p* = 0.0003).

**Figure 4:**
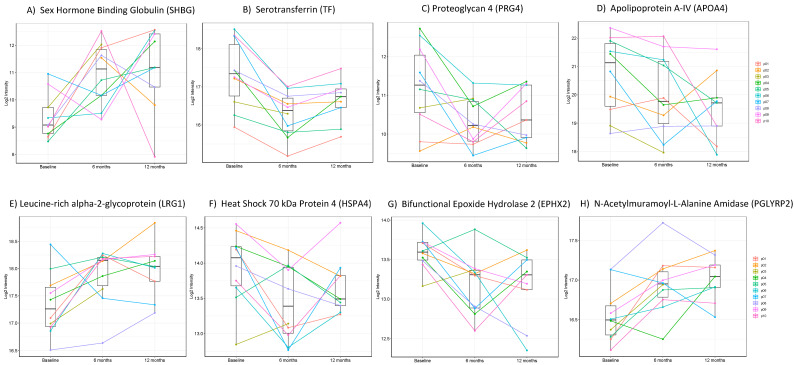
(**A**–**H**): Changes in protein expression between baseline, 6 months and 12 months for the 8 differentially expressed proteins at both 6 months and 12 months post-surgery.

The relative quantification information acquired using SWATH-MS for 467 proteins in a longitudinal fashion has a degree of power as temporal analyses in humans provide greater surety of biomarker status. Relative to pre-surgery samples, eight proteins were significantly different at both 6- and 12-months post-surgery (Figure 4). Of those eight proteins, three showed increased expression after BS (FC: fold change baseline vs. 12 months), *p* value); sex hormone binding globulin (SHBG) (1.95, *p* < 0.01), leucine-rich alpha-2-glycoprotein (LRG1) (0.59, *p* < 0.05) and N-acetylmuramoyl-L-alanine amidase (PGLYRP2) (0.43, *p* < 0.05), whilst the remaining five showed decreased expression; serotranferrin (TF) (−0.78, *p* < 0.01), proteoglycan 4 (PRG4) (−0.78, *p* < 0.05), Apolipoprotein A4 (APOA4) (−1.38, *p* < 0.05), heat shock protein 4 (HSPA4) (−0.38, *p* < 0.05), bifunctional epoxide hydrolase 2 (EPHX2) (Table 4). The greatest fold change was seen for SHBG approaching a two-fold elevation after BS. The correspondent protein names and gene symbol for each code used to build the figures (volcano plots and heatmaps) can be found in the supplementary material.

## 5. Discussion

Using the technique of SWATH-MS to generate proteomic maps, we have shown it is possible to separate samples collected pre- and post bariatric surgery on the basis of the plasma proteome with data from a relatively small sample size. We found a significant change in plasma protein levels for a number of metabolically relevant proteins from pre-BS to 6- and 12-months post-surgery with eight proteins showing change at both 6- and 12-months vs. pre-surgery levels. The relevant proteins common to both post-surgery periods analysed are TF, APOA4, HSPA4, LRG1, PGLYRP2, SHBG, EPHX2 and PRG4.

Greater resolution between baseline and post-bariatric surgery samples was noted when proteins with significant fold changes were solely included in the analysis, as a means of discovering potential biomarkers; Figure 2 shows the PCA of the significant proteins between baseline and 12 months and Figure 3 shows the same between baseline and 6 months. The clearest separation occurs between baseline and 12 months which coincides with the onset of the time period at which often the greatest weight loss is observed with BS [25]. We previously described proteomic changes in relation to a lifestyle change intervention in non-diabetic hyperglycaemia and also showed that the baseline levels of certain proteins such as insulin-like growth factor (IGF)-II and vitamin D binding protein were predictive of more or less weight loss with that lifestyle change intervention [26].

Previous work employing SWATH-MS to study biomarkers in patients with impaired glucose tolerance who lost weight in response to a diet and exercise programme and in whom HbA1c resolved identified the following major differentiating proteins: Insulin-like growth factor 2 (IGF-II), Retinol binding protein 4 (RBP4), Fetuin-A (FetA) -Zinc-α2- glycoprotein (ZA2G), Visfatin (NAMPT), fatty acid synthase (FAS) and vitamin D binding protein (VDR) [26]. The disparity between this study and ours suggests a differential process and a potentially specific set of biomarkers for remission of T2DM in patients undergoing BS compared to those achieving weight loss through other structured means. No difference in circulating IGF binding proteins was picked up by the SWATH-MS analysis in the present study. HOMA2-IR as a measure of insulin resistance decreased and HOMA2-B as measure of pancreatic beta cell function increased numerically; this did not reach statistical significance.

Previously Varela-Rodríguez et al. showed, using proteomic analysis, that BS remodels subcutaneous adipose tissue function to influence specific molecular mechanisms with lower inflammation, increased uptake of glucose, higher insulin sensitivity, higher de novo lipogenesis, increased mitochondrial function and decreased adipocyte size [27]. Our results support these findings as the cluster of relevant proteins showing a change after 6 months of BS are involved in biological process such as protein-lipid remodelling, leukocyte mediate immunity and response to stimulus pathways; and after 12 months, proteins involved in healing, blood coagulation and immune activation.

The panel of eight proteins identified as discriminating between the groups, and common at both 6- and 12-months post-surgery, included proteins known to be related to insulin sensitivity and glucose intolerance (TF, SHBG and PRG4) [28,29,30], relate to systemic inflammation (HSPA4 and APOA4) [31,32] ,also those involved in the immune response (LRG1 and PGLYRP2) [33,34,35,36] and cholesterol function (EPHX2) [37,38]. We have highlighted the importance of each protein in the context of obesity, BS and T2DM below.

LRG1 is a circulating protein first discovered in 1977 [39] whose function has remained elusive for many years. Some evidence has suggested that it may function, in part, as an angiogenesis factor [40]. It has been observed as a marker of inflammation in the sputum of asthmatic patients [41], in the serum of patients with ulcerative colitis [42] and autoimmune disease respectively [43]. Shirai et al. showed that it is often up-regulated during the acute phase response [44]. Studies have also found LRG1 to be higher in patients with T2DM and peripheral arterial disease [45]. Notwithstanding previous studies, our results demonstrate a clear increase in LRG1 expression both 6- and 12-months after surgery. This is a similar finding to that of Roriguez-Rivera et al. [46] one year after surgery and Pek et al. who prospectively studied 231 morbidly obese patients undergoing BS and found LRG1 levels to significantly increase just 1.5 months after surgery [47]. These results suggest that inflammation is probably not the sole inducer of LRG1 expression and further research into the role of this glycoprotein is required.

TF levels declined after BS surgery in our cohort, which may reflect the reduced inflammatory state manifested in tandem with weight loss after BS. TF is a part of the innate immune system and acts as a marker of inflammation in patients with T2DM [48]. Furthermore, pre-operative deficiencies of iron and other trace elements are prevalent amongst individuals with obesity undergoing surgery and are often replaced post-surgery [49], which offers an explanation as to the maintained significant decline in this protein.

HSPA4 is largely thought to function as a cytosolic chaperone involved in facilitating protein folding, degradation, complex assembly and translocation. Significantly higher levels of HSPA4 have been reported in T2DM of longer duration compared to newly diagnosed [50]. Nakhiayani et al. showed that HSPA4 was associated with the inhibition of nitric oxide production in individuals with T2DM [51], confirmed that it correlated with CRP [51] whilst Morteza et al. found that the odds ratio for its predictive value for micro-albuminuria in T2DM was highly significant [52]. Garamyolgyi et al. showed that HSPA4 levels correlated well with HbA1c in gestational diabetes [53]. Our work demonstrates a significant reduction in HSPA4 which correlated with percentage change in IL6 between baseline and 12 months (r = 0.87, *p* < 0.001), this is in keeping with previous work by Styger et al. who demonstrated reduction in both serum and liver HSPA4 in a murine model after BS [54] and also is in agreement with the narrative that HSPA4 appears to be induced by states of chronic inflammation [55]. Some have suggested targeting these proteins as therapy for T2DM [56].

In our cohort, APOA4 was noted to significantly and consistently reduce at both time points after BS which may appear intuitive since this lipid-binding protein is primarily synthesized in the small intestine, which is bypassed during this type of surgery [57]. In animal models circulating Apoa4 appear to confer some protection against diabetes [58,59] and atherosclerosis. However, its role in humans has not fully been delineated. Previous studies conflict in their results surrounding this lipoprotein with some reporting no change after BS [60] whilst others have increases in APOA4 levels post-surgery [46,61]. Rao et al. reported that BS resulted in a decrease in HOMA-IR in patients after RYGB and higher baseline APOA4 levels correlated with the decrease in HOMA-IR. Another recent study, utilizing proteomic methods, analysed a large number of obese individuals who were subject to low calorie diet finding that, amongst others, APO A4 levels declined after the eight week intervention [62]. We speculate that APOA4 may be a physiologic compensation to insulin resistance that resolves after BS. In the circulation, APOA4 is present on chylomicron remnants, high-density lipoproteins, and in lipid-free form. APOA4 is involved in a myriad of physiological processes such as lipid absorption and metabolism, anti-atherosclerosis, platelet aggregation and thrombosis, glucose homeostasis and food intake. APOA4 deficiency is associated with atherosclerosis and diabetes, which renders it as a potential therapeutic target for treatment of these diseases [63].

PGLRYP2 [64] is an enzyme that breaks down glycopeptides and can break down the cell walls of bacteria [65] giving it a principal role in fighting bacterial infection. It is expressed constitutively from hepatocytes [66] and proteomic studies have previously suggested a possible utility in diagnosing sepsis in critically ill patients [67]. Expression can be induced in skin keratinocytes after exposure to certain bacteria [68] as well as in intestinal and oral epithelial cells [69]. Our study demonstrates a significant increase in fold change of this protein at both 6- and 12-months post-surgery with correlations with percentage change in BMI at 6 months (*p* < 0.05) but not 12 months. We speculate that this change may result from the resolution of non-alcoholic hepatic steatosis (NAHS) in our patients. Previous work has shown that the PGLRYP2 gene is hypermethylated in patients with NAHS [70] and immune-competent mice who overexpress PGLRYP2 display significantly enhanced anti-cancer immune responses against hepatocellular carcinoma (HCC) [35]. Indeed, resolution of both NAHS and lower incidence of HCC are recorded as one of the beneficial effects of BS [71,72].

SHBG is produced and secreted by the liver and acts to regulate the bioavailability of sex steroids. [73]. Low serum SHBG concentrations are associated with metabolic syndrome [74], T2DM [75] and increased risk for cardiovascular problems. [76] In obesity, SHBG levels are low and associated with pro-inflammatory cytokines and hepatic steatosis [74]. Our results showed an increase in SHBG levels after BS consistent with results found previously in men and women after BS [77,78].

EPHX2 is a soluble enzyme that hydrolyses epoxyeicoasatrienoic acids (EETs) to their inactive diols [79] EETS play important roles in vasodilation [80], lipid metabolism [81] improve insulin sensitivity [82] and have both anti-inflammatory [83] and analgesic properties [84]. Gene deletion and pharmacological inhibition of EPHX2 exhibit an increase in insulin sensitivity in a T2DM rodent model [85]. Furthermore, its variants are likely to be associated with neuropathy in T2DM patients [86]. EPHX2 circulating levels are elevated in obese individuals and its expression is attenuated after physical activity in both adipose tissue and peripheral blood mononuclear cells [87]. Higher EPHX2 levels are significantly associated with risk of incident CVD [88]. We observed a consistent reduction of EPHX2 at both 6- and 12- months post-BS. The inhibition of EPHX2 has been suggested as previous therapeutic target to tackle CVD and this requires further study [89].

PRG4 was first identified as a component of the extracellular matrix in synovial fluid implicated in shear force reduction in cartilage [90]. However, recent evidence suggests that it can act as a “buffer” by effecting downstream signalling during an inflammatory response [91]. Furthermore, chronic inflammatory states cause post-transcriptional modifications resulting in dysfunctional PRG4 upsetting homeostasis [92]. PRG4 levels declined significantly at both time points which is a consistent finding by previous groups studying the effect of weight loss on the proteome [93,94] and has previously been found to correlate with insulin sensitivity [94]. Knockout PRG4 mice exhibit better glucose handling when fed a high-fat diet and are protected from hepatic steatosis and white adipose tissue inflammation [30]. The regulation and roles of PRG4 in obesity are largely unknown and require further work to be delineated.

## 6. Strengths and Limitations

We are showing here preliminary data from a hypothesis-driven study. Our study utilises data on only 10 people, mostly female, which is a limitation. Nevertheless, we have described significant changes in the proteome in relation to remission of T2DM. We have identified significant differences in the plasma levels of a number of metabolically relevant proteins after BS in relation to remission of T2DM using a technique that has high reproducibility and sensitivity, in three time points (baseline, 6- and 12-months post-surgery). In this respect, it offers a potential set of biomarkers for remission that require further investigation, which is now planned. An additional limitation is that we have only here described changes in protein levels in plasma rather than in adipose or other tissues. However, biomarkers are generally measured in biofluids. Finally, our study did not compare remitters with non-remitters, which would add further value in identifying biomarkers relevant to T2DM remission. Further analysis using different and larger cohorts are necessary to replicate our findings.

## 7. Conclusions

Using SWATH-MS we found significant changes in the proteome for eight proteins from pre-BS to 6/12 and 12/12 post-BS. This panel of proteins identified as consistently different, included peptides related to insulin sensitivity (SHBG increase), systemic inflammation (TF and HSPA4—both decreased) and lipid metabolism (APOA4 decreased). Several of these are key components in metabolic, immune system and inflammatory pathways. Therefore, these protein changes merit further exploration as potential marker signatures in patients who undergo remission of T2DM.

## Figures and Tables

**Table 1:** Anthropometric and clinical measurements taken from patients before and after bariatric surgery.

	Baseline	6 Months	12 Months	*p*-Value
Number of samples	10	10	10	
Age (years)	53 CI (46–60)			
Sex	2 Males, 8 Females			
**Anthropometric and Clinical** *				
Height (cm)	Male: 177 Female: 163 cm			
BMI (kg/m^2^)	54.9 (10.3)	41.8 (6.96) *	38.6 (5.08) * ^†^	*p* < 0.0001
Systolic BP	135 (22.6)	123 (16.7)	116 (14.4) *	*p* < 0.05
Diastolic BP	79.2 (17.3)	67.9 (14.1)	67.9 (11) *	*p* < 0.05
**Biochemical**				
HbA1c (mmol/mol)	56.0 (14.1)	40.0 (5.4) *	38.2 (6.25) * ^†^	*p* < 0.0001
Fasting plasma glucose	7.9 (3.5)	5.8 (2.0)	4.8 (0.70)	*p* < 0.05
HOMA2-IR	0.57 (0.52)	0.489 (0.6)	0.34 (0.24)	*p* = 0.57
HOMA2-B (%)	28.9 (18.2)	45.5 (32.7)	48.9 (16.7)	*p* = 0.14

* Significant compares to baseline; ^†^ significant compared to 6 months; data are presented as mean (standard deviation); CI: confidence interval.

**Table 2:** Proteins showing a significant difference in expression between baseline and six months post-surgery.

Protein Names (Gene Symbol)	Fold Change (Log2)	*p* Value
Serotransferrin (TF)	−1.068	<0.0001
Beta-Ala-His dipeptidase (CNDP1)	−0.952	0.006
Complement C3 (C3)	−0.555	0.008
Proteoglycan 4 (PRG4)	−0.898	0.011
Cholinesterase (BCHE)	−0.581	0.012
Sex hormone-binding globulin (SHBG)	1.485	0.013
Apolipoprotein M (APOM)	0.689	0.018
Protein disulfide-isomerase A3 (PDIA3)	1.527	0.021
Bifunctional epoxide hydrolase 2 (EPHX2)	−0.412	0.022
Apolipoprotein A-IV (APOA4)	−0.713	0.023
N-acetylmuramoyl-L-alanine amidase (PGLYRP)	0.391	0.024
Angiotensinogen (AGT)	−1.036	0.025
T-complex protein 1 subunit theta (CCT8)	−1.235	0.026
Heat shock 70 kDa protein 4 (HSPA4)	−0.497	0.026
Leucine-rich alpha-2-glycoprotein (LRG1)	0.532	0.032
Pigment epithelium-derived factor (SERPINF1)	−0.391	0.032
C4b-binding protein alpha chain (C4BPA)	−0.393	0.032
Exportin-1 (XPO1)	−0.746	0.033
Immunoglobulin heavy constant mu (IGHM)	−0.978	0.034
Alpha-1-acid glycoprotein 2 (ORM2)	−0.742	0.036
Sulfhydryl oxidase 1 (QSOX1)	−0.775	0.037
Apolipoprotein C-III (APOC3)	−1.171	0.039
Basement membrane-specific heparan sulfate proteoglycan core protein (HSPG2)	−0.435	0.041
Heparin cofactor 2 (SERPIND1)	−0.415	0.042
Aminopeptidase (ANPEP)	−0.395	0.048

**Table 3:** Proteins showing a significant difference in expression between baseline and 12 months post-surgery.

Protein Names (Gene Symbol)	Fold Change (log2)	*p* Value
Serum amyloid *p*-component (SAP)	−0.936	0.001
Apolipoprotein A-I (APOA1)	1.104	0.001
Serotransferrin (TF)	−0.776	0.002
Inter-alpha-trypsin inhibitor heavy chain H3 (ITIH3)	0.799	0.002
Fibronectin (FN1)	−1.724	0.003
Sex hormone-binding globulin (SHBG)	1.951	0.003
Neural cell adhesion molecule L1-like protein (L1CAM)	0.663	0.007
Haptoglobin (HP)	−1.383	0.007
Apolipoprotein A-IV (APOA4)	−1.381	0.008
Antithrombin-III (SERPINC1)	0.541	0.008
Peptidase inhibitor 16 (PI16)	0.742	0.009
Galectin-3-binding protein (LGALS3BP)	−0.590	0.009
Laminin subunit gamma-1 (LAMC1)	−1.506	0.012
Proteoglycan 4 (PRG4)	−0.779	0.015
Hemoglobin subunit delta (HBD)	−1.088	0.020
**N-acetylmuramoyl-L-alanine amidase (PGLYRP2)**	0.438	0.021
Dermcidin (DCD)	0.905	0.021
Heat shock 70 kDa protein 4 (HSPA4)	−0.389	0.022
Actin-related protein 2/3 complex subunit 1B (ARPC1B)	−0.403	0.023
Bifunctional epoxide hydrolase 2 (EPHX2)	−0.473	0.025
Hyaluronan-binding protein 2 (HABP2)	0.358	0.025
Galactokinase (GALK1)	0.899	0.027
Alpha-2-macroglobulin (A2M)	0.801	0.027
Leucine-rich alpha-2-glycoprotein (LRG1)	0.591	0.027
Peroxiredoxin-6 (PRDX6)	0.627	0.028
Thyroxine-binding globulin (SERPINA7)	0.365	0.030
Mitogen-activated protein 3 kinase 5 (MAP3K5)	0.632	0.031
Alpha-1B-glycoprotein (A1BG)	0.462	0.033
Complement factor H (CFH)	−0.415	0.036
Protein AMBP (AMBP)	0.352	0.037
L-lactate dehydrogenase A chain (LDHA)	−1.538	0.037
Endoplasmin (HSP90B1)	−0.417	0.038
Angiotensin-converting enzyme (ACE)	−0.405	0.039
**Plasma protease C1 inhibitor (SERPING1)**	0.939	0.039
Desmin (DES)	−0.952	0.043
Vitamin D-binding protein (GC)	0.381	0.046
Secreted phosphoprotein 24 (SPP2)	0.475	0.048
T-complex protein 1 subunit gamma (CCT3)	−0.722	0.049
Haemoglobin subunit beta (HBB)	−1.128	0.049

**Table 4:** Proteins showing a significant difference in expression at both 6- and 12-months post-surgery. Log fold changes are shown between baseline and 12 months.

Protein Names (Gene Symbol)	Fold Change (log2)	*p* Value
Serotransferrin (TF)	−0.776	0.002
Apolipoprotein A-IV (APOA4)	−1.381	0.008
Heat shock 70 kDa protein 4 (HSPA4)	−0.389	0.022
Leucine-rich alpha-2-glycoprotein (LRG1)	0.591	0.027
**N-acetylmuramoyl-L-alanine amidase (PGLYRP2)**	0.438	0.021
Sex hormone-binding globulin (SHBG)	1.951	0.003
Bifunctional epoxide hydrolase 2 (EPHX2)	−0.473	0.025
Proteoglycan 4 (PRG4)	−0.779	0.015

## Data Availability

Any requests for data extracts will be considered by Adrian Heald.

## Supplementary Materials

The following are available online at https://www.mdpi.com/article/10.3390/jcm10163659/s1.

## Abbreviations

A2M	Alpha-2-macroglobulin
AGT	Angiotensinogen
APOA1	Apolipoprotein A1
APOA4	Apolipoprotein A4
APOC3	Apolipoprotein C3
APOM	Apolipoprotein M
BMI	Body Mass Index
BS	Bariatric Surgery
CRP	C—reactive protein
CVD	Cardiovascular disease
EETs	Epoxyeicoasatrienoic acids
EPHX2	Bifunctional epoxide hydrolase 2
FE	Fold Enrichment
FN1	Fibronectin
GO	Gene Ontology
HbA1c	glycosylated haemoglobin
HCC	Hepatocellular carcinoma
HOMA-B	Homeostatic model assessment of beta cell function
HOMA-IR	Homeostatic model assessment of insulin resistance
HP	Haptoglobulin
HSPA4	Heat shock 70 kDa protein 4
IGF-II	Insulin-like growth factor II
LC-MS	Liquid Chromatography Mass spectrometry
LRG1	Leucine-rich alpha-2-glycoprotein
NAHS	Non-Alcoholic Hepatic Steatosis
PCA	Principal component analysis
PGLYRP2	N-acetylmuramoyl-L-alanine amidase
PRG4	Proteoglycan 4
SERPINC1	Antithrombin-III
SERPING1	Plasma protease C1 inhibitor
SHBG	Sex Hormone Binding Globulin
SWATH-MS	Sequential window acquisition of all theoretical fragment ion spectra Mass Spectrometry
T2DM	Type 2 Diabetes Mellitus
TF	Serotransferrin
